# Priority setting for children and young people with chronic conditions and disabilities

**DOI:** 10.1111/hex.13761

**Published:** 2023-04-20

**Authors:** Amy Finlay‐Jones, Rebecca Sampson, Asha Parkinson, Karina Prentice, Keely Bebbington, Claire Treadgold, Belinda Frank, Amber Bates, Jacinta Freeman, Jayden Lucas, Julie Dart, Elizabeth Davis, Raghu Lingam, Anne McKenzie

**Affiliations:** ^1^ Early Neurodevelopment and Mental Health Telethon Kids Institute Nedlands Western Australia Australia; ^2^ School of Population Health Curtin University Perth Western Australia Australia; ^3^ Medical School University of Western Australia Perth Western Australia Australia; ^4^ Starlight Children's Foundation Naremburn New South Wales Australia; ^5^ University of New South Wales Sydney New South Wales Australia; ^6^ Tiny Sparks WA West Leederville Western Australia Australia; ^7^ Perth Children's Hospital Nedlands Western Australia Australia

**Keywords:** child and adolescent, chronic conditions, consumer involvement, disability, priority setting

## Abstract

**Background:**

The aim of this project was to identify the top 10 priorities for childhood chronic conditions and disability (CCD) research from the perspectives of children and young people with lived experience, their parents and caregivers and the professionals who work with them.

**Methods:**

We conducted a three‐stage study based on the James Lind Alliance priority‐setting partnership methods. It comprised two online surveys (*n* = 200; *n* = 201) and a consensus workshop (*n* = 21) with these three stakeholder groups in Australia.

**Results:**

In the first stage, 456 responses were submitted, which were coded and collapsed into 40 overarching themes. In the second stage, 20 themes were shortlisted, which were further refined in stage 3, before the top 10 priorities being selected. Of these, the top three priorities were improving awareness and inclusion in all aspects of their life (school, work and social relationships), improving access to treatments and support and improving the process of diagnosis.

**Conclusions:**

The top 10 priorities identified reflect the need to focus on the individual, health systems and social aspects of the CCD experience when conducting research in this area.

**Patient or Public Contribution:**

This study was guided by three Advisory Groups, comprising (1) young people living with CCD; (2) parents and caregivers of a child or young person with CCD and (3) professionals working with children and young people with CCD. These groups met several times across the course of the project and provided input into study aims, materials, methods and data interpretation and reporting. Additionally, the lead author and seven members of the author group have lived and experienced CCD.

## 1 BACKGROUND

Chronic conditions and disabilities (CCD) affect at least 10% of children and young people worldwide, although some studies estimate a much higher prevalence,^1^, ^2^ with prevalence for specific conditions varying across age bands.^3^ Living with a CCD can have profound and pervasive impacts on children and their families, including 2–3 times greater odds of academic underperformance,^4^ double the likelihood of experiencing psychological distress^5^ and disruptions across social, family and financial domains.^6^ Further, children and young people with CCD often experience complicated and lengthy pathways to diagnosis and support due to a lack of health literacy and limited health provider understanding coupled with stigmatising attitudes and beliefs.^7^ Given the increasing prevalence of these conditions and their pervasive impacts, it is critical that more research is directed towards promoting meaningful outcomes amongst these individuals and their families.

A starting point to achieving this is asking people with lived experience of CCD which research topics are most meaningful to them. Researchers and decision‐makers are increasingly valuing stakeholder involvement in defining research priorities due to the many benefits presented.^8^, ^9^, ^10^ Stakeholder involvement promotes positive impacts across the entire research and translation pipeline, including the development of user‐relevant research questions, greater engagement of research participants and more efficient and consumer‐relevant implementation, dissemination and translation of evidence into policy and practice.^8^, ^11^ Despite these benefits, research questions and directions defined by researchers do not always reflect the priorities of patients, their families and service providers.^12^, ^13^, ^14^ A previous review of 258 research priority‐setting studies found that only 19% of studies involved both patients and clinicians,^15^ while a review of 83 paediatric chronic disease priority‐setting studies found that only 4% involved patients.^16^

Odgers et al.^16^ recommended that priority‐setting for paediatric chronic disease follow a systematic and explicit process to develop a translational research agenda that is meaningful for patients, parents, clinicians and researchers alike. A methodology known as a ‘priority‐setting partnership’ (PSP) has been developed by the James Lind Alliance (JLA) for identifying and prioritising health research questions that matter to patients, their families and clinicians.^17^ PSPs bring key stakeholders together to determine the most important unanswered research questions concerning a particular patient group or groups. PSPs have been used to identify stakeholder priorities for research on specific long‐term conditions in childhood, including children with neurodisability,^18^ cancer,^19^, ^20^ asthma,^21^ cleft lip and palate,^22^ eczema,^23^ cystic fibrosis^24^ and chronic pain.^25^ Prior PSPs have revealed the need for research agendas to encompass a more holistic approach to supporting outcomes for children, young people and families, rather than being restricted to biological and drug intervention research.

These recommendations were echoed in recent work with paediatric chronic conditions groups in Australia^26^ and Canada.^27^ These studies were valuable in their transdiagnostic approach: many young people with CCD experience significant comorbidity and identifying common priorities can help to improve efficiency and collaboration in research. However, one study^26^ only involved a small number of children and young people (*n* = 3) and parents (*n* = 19) relative to professionals (*n* = 51), while in the other,^27^ only 22%–29% of the sample had lived experience. Accordingly, the aim of the current research was to build on this work by seeking input from a larger proportion of young people and parents/caregivers from across Australia. Our goal was to identify the top 10 priorities for research on child and adolescent CCD, from the perspective of young people with CCD, their parents and caregivers and the professionals who work with them.

## 2 METHODS

### 2.1 Participants

This study recruited Australian participants from three groups: (1) Young people aged 14–25 years with CCD; (2) parents and caregivers with a child under the age of 25 years with CCD and (3) professionals working with young people aged 0–25 years of age with CCD. There were no exclusion criteria regarding types of CCD.

### 2.2 Procedure

**Figure 1:**
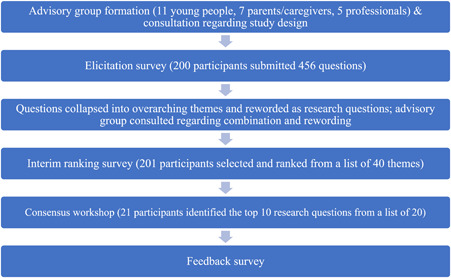
Overview of the child and adolescent chronic conditions and disability priority‐setting process.

The WA Health Child and Adolescent Health Service Ethics Committee approved the project (RGS0000003842), and all participants provided informed consent to participate. Guided by an abridged version of the JLA methodology,^28^, ^29^ the project involved (1) forming Advisory Groups; (2) conducting a preliminary survey to elicit initial questions; (3) grouping and coding the initial questions into themes; (4) conducting a second survey to rank the themes and (5) an online workshop to reach consensus on a top‐10 list of priorities for future research. An overview of the study design is shown in Figure 1.

### 2.3 Advisory group formation

We recruited three Advisory Groups, comprising (1) 11 young people with CCD; (2) seven parents and caregivers of young people with CCD and (3) five professionals in clinical, advocacy or decision‐making roles in the child and adolescent CCD field. Advisory Groups met at least twice across the course of the project to discuss the project scope, provide feedback on project methods and support recruitment and translation strategies. Additionally, the research team was led by a researcher with CCD, supported by two research assistants and three workshop facilitators with lived experience of CCD.

### 2.4 Recruitment

At each stage, participants were recruited via social media and through mailing lists of CCD advocacy agencies and community support groups. Participants who consented to be contacted about the project were emailed as each stage became active. Participants were not required to have been involved in previous stages to join, with additional participants recruited in each stage.

#### 2.4.1 Elicitation survey

An initial online survey was conducted to elicit participants' questions related to child and adolescent CCD that they would like answered by research. Demographic data included postcode, gender, age, Aboriginal and/or Torres Strait Islander status, CCD diagnosis and duration of CCD. Young people and parents were asked, ‘Please tell us your most important questions, needs, or concerns about [your/your child's] chronic condition/s and/or disabilities. These can be concerns that you would like to ask your healthcare provider about or questions that you think are important for researchers to study’. Professionals were asked, ‘From your perspective, what are the most important unanswered questions about the impact of childhood chronic illness and disability on children and young people? These might be questions you are frequently asked by young people or their families, or other questions that you believe should be the subject of future research’.

Following this open‐ended question, participants were prompted to consider whether they had specific questions related to school and learning; relationships; well‐being and mental health; promoting acceptance and understanding; patient and family education; condition management; transition out of child and adolescent services; supporting parents and families; accessing or navigating treatment or support and the future. These prompts were developed in consultation with our Advisory Groups. Participants were then asked to list any other questions that did not fit under a previous heading.

We conducted a content analysis of responses using NVivo.^30^ After familiarisation with the data, we conducted primary coding of each question to identify manifest content. Secondary coding was conducted to identify higher‐level meanings that could be extracted from each question. This enabled questions to be combined into themes that could be summarised by a single overarching question. Input from the Advisory Groups was sought regarding the phrasing of the themes and to determine whether these adequately captured the content provided from stage 1. Where survey responses were not framed as questions, they were reframed following dialogue between two of the researchers (A. F.‐J‐ and B. S.) and the Advisory Groups.

#### 2.4.2 Ranking survey

A second online survey was conducted to establish a shortlist of themes to take to the consensus workshop. Participants were presented with the list of themes following stage 1 (40 in total) and asked to select as many themes as they felt were important to them. Their selections were carried through to the second step, in which participants were asked to rank questions in order from most to least important. Data were downloaded to SPSS to identify the top 20 most frequently selected and highly ranked questions.

#### 2.4.3 Online consensus workshop

The final stage of the project involved a 2.5‐h online workshop. A summary of the previous stages of the project and an explanation of the aim of the workshop were provided to the group. Participants were divided into smaller ‘breakout’ groups, comprising a mix of young people, professionals and parents. Each group was facilitated by a member of the research team with experience in the JLA priority‐setting methodology and small‐group facilitation. Groups had access to their own Google Jamboard, which displayed the top 20 themes on digital sticky notes that could be modified and moved during the discussion.

In the first breakout group session, facilitators led the groups through each theme sequentially, asking for input regarding the wording of the theme, whether themes could be combined further or if any important themes were missing. Following this, all participants returned to the main room, and facilitators were asked to present the outcome of these discussions on behalf of their group. As a whole group, themes were reworded, and a consensus was reached on themes that could be combined. The consensus was considered to have occurred when, following a period of discussion, no further comments or requests for the amendment were put forward after appropriate prompts. In a second breakout group session, facilitators led each group in arranging themes from most to least important. The top 10 priorities were then determined by scoring each question according to its ranking in each group's list. Participants were also asked to provide feedback on the workshop process and top 10 priorities. Specifically, they were asked to identify whether there were any groups that the priorities may not be relevant to and whether there were any ideas for how research might address the top‐ranked priority.

## 3 RESULTS

Two‐hundred participants (94 young people, 78 parents and 28 professionals) took part in stage 1 and 201 participants (73 young people, 95 parents and 33 professionals) took part in stage 2. The workshop was attended by 21 participants, including 12 young people, 3 parents and 6 professionals. Demographics and clinical characteristics of the sample who participated in stages 1 and 2 are reported in Tables 1 and 2. ‘Other’ conditions reported by young people and parents/caregivers at stages 1 and 2 included 81 conditions listed in Table 3. Professionals were from a range of backgrounds, including occupational therapists, psychologists, rare disease advocates, general paediatricians, paediatric neurologists, paediatric oncologists, nurses, physiotherapists, in‐hospital children's entertainers and representatives from CCD advocacy groups.

**Table 1:** Demographic and clinical characteristics of the sample in stage 1.

	**Young person (*n*** = **94)**	**Parents/caregivers (*n*** = **78)**	**Professionals (*n*** = **28)**
Gender			
Male	29 (30.8%)	5 (6.4%)	8 (28.6%)
Female	62 (66.0%)	72 (92.3%)	20 (71.4%)
Nonbinary	3 (3.2%)	1 (1.3%)	0 (0.00%)
Aboriginal and/or Torres Strait Islander			
Yes	2	2	1
No	92	76	27
State			
ACT	3 (3.2%)	1 (1.3%)	0
NSW	19 (20.2%)	11 (14.1%)	7 (25%)
NT	2 (2.1%)	2 (2.6%)	2 (7.1%)
QLD	4 (4.3%)	5 (6.4%)	0
SA	5 (5.3%)	1 (1.3%)	0
TAS	2 (2.1%)	2 (2.6%)	2 (7.1%)
VIC	10 (10.6%)	11 (14.1%)	5 (17.9%)
WA	49 (52.1%)	45 (57.7%)	12 (42.9%)

Proportion of sample diagnosed with or working with specific chronic conditions			
	**Young person self‐report (*n*** = **94** a **)**	**Parent report about the child (*n*** = **78** a **)**	**Professional focus area (*n*** = **28** a **)**
Asthma	17 (18.1%)	5 (6.4%)	7 (25.0%)
Allergies	22 (23.4%)	12 (15.4%)	5 (17.9%)
Burns	8 (8.5%)	1 (1.3%)	3 (10.7%)
Cancer	11 (11.7%)	8 (10.3%)	7 (25.0%)
Diabetes	23 (24.5%)	6 (7.7%)	8 (28.6%)
Eczema	0 (0.00%)	1 (1.3%)	3 (10.7%)
Endometriosis	19 (20.2%)	1 (1.3%)	2 (7.1%)
IBD	21 (22.3%)	5 (6.4%)	7 (25.0%)
ME/CFS	22 (23.4%)	11 (14.1%)	8 (28.6%)
Multiple sclerosis	1 (1.1%)	0 (0.00%)	1 (3.57%)
Other	30 (31.9%)	32 (41.02%)	18 (64.3%)

Abbreviations: CFS, chronic fatigue syndrome; IBD, inflammatory bowel disease; ME, myalgic encephalomyelitis.

^a^ Note that some respondents reported more than one condition; proportions are calculated based on the total n for each participant category, but cumulative proportions do not sum to 100.

**Table 2:** Demographic and clinical characteristics of the sample in stage 2.

	Young person	Parents/caregivers	Professionals
	(*n* = 73)	(*n* = 95)	(*n* = 33)
Gender			
Male	24 (32.9%)	7 (7.4%)	8 (24.2%)
Female	32 (43.8%)	61 (64.2%)	24 (72.7%)
Nonbinary	12 (16.4%)	0 (0.0%)	0 (0.0%)
Not reported	5 (6.8%)	27 (28.4%)	1
Child age (years)			
Mean (SD)	21.9 (2.7)	11.65 (6.04)	
Range	16‐25	0‐25	
Aboriginal and/or Torres Strait Islander			
Yes	2 (2.7%)	4 (4.2%)	3 (9.1%)
No	69 (94.5%)	91 (95.8%)	25 (75.8%)
NR	2 (2.7%)		5 (15.1%)
State			
ACT	4 (5.5%)	2 (2.1%)	1 (3.0%)
NSW	17 (23.3%)	20 (21.1%)	14 (42.4%%)
NT	2 (2.7%)	2 (2.1%)	1 (3.0%)
QLD	7 (9.6%)	10 (10.5%)	2 (6.0%)
SA	5 (6.8%)	5 (5.3%)	3 (9.1%)
TAS	1 (1.4%)	2 (2.1%)	1 (3.0%)
VIC	8 (10.9%)	7 (7.4%)	4 (12.1%)
WA	29 (39.7%)	47 (49.5%)	6 (18.2%)

The proportion of the sample diagnosed with or working with specific chronic conditions			
	**Young person self‐report (*n*** = **73** a **)**	**Parent report about the child (*n*** = **95** a **)**	**Professional focus area (*n*** = **33** a **)**
Asthma	17 (23.3%)	11 (11.6%)	7 (21.1%)
Allergies	16 (21.9%)	15 (15.8%)	4 (12.1%)
Burns	0 (0.0%)	0 (0.0%)	1 (3.0%)
Cancer	0 (0.0%)	3 (3.2%)	7 (21.2%)
Diabetes	7 (9.6%)	2 (2.1%)	6 (18.2%)
Eczema	9 (12.3%)	9 (9.5%)	3 (9.1%)
Endometriosis	13 (17.8%)	0 (0.0%)	3 (9.1%)
IBD	5 (6.8%)	0 (0.0%)	4 (12.1%)
ME/CFS	13 (17.8%)	2 (2.1%)	4 (12.1%)
Multiple sclerosis	0 (0.0%)	0 (0.0%)	3 (9.1%)
Other	33 (45.2%)	59 (62.1%)	15 (45.5%)

Abbreviations: CFS, chronic fatigue syndrome; IBD, inflammatory bowel disease; ME, myalgic encephalomyelitis.

^a^ Note that some respondents reported more than one condition; proportions are calculated based on the total *n* for each participant category, but cumulative proportions do not sum to 100.

**Table 3:** List of ‘other’ conditions reported by young people and parents/caregivers at stages 1 and 2.

Condition	S1	S2	Condition	S1	S2
Acquired brain injury	1	1	Gastroparesis	2	4
Addison's disease	1	1	Granulomatosis with polyangiitis	2	1
Adenomyosis	1	1	Haemophilia	–	1
Ankylosing spondylitis	2	2	Hearing loss and deafness	–	3
Anorexia nervosa	1	–	Hypermobility syndrome	2	2
Amputee	–	1	Hypermyelinating white matter disorder	–	1
Apnoea	–	1	Hypothyroidism	–	2
Arthrogryposis	–	1	Idiopathic hypersomnia	–	2
Autism spectrum disorder	4	12	igG4‐sclerosing cholangitis	1	1
Autonomic dysreflexia		3	Intellectual disability	1	4
ARFID	–	1	Intradural Spinal Lipoma	1	
Batten disease	–	1	Juvenile arthritis	3	5
Bladder dysfunction	–	2	Lissencephaly	–	1
Bradycardia	–	1	Liver transplant	–	1
Brain stem dysfunction	–	1	Lowe's syndrome	1	1
Cerebral palsy	3	4	Lupus	–	2
Childhood dementia	1	1	Mannose‐binding lectin deficiency	–	1
Chronic interstitial lung disease	–	1	Marfan syndrome	–	1
Cirrhosis	–	1	Mast cell activation syndrome	–	1
Cloacal anomaly	–	2	Metabolic condition	–	1
Coeliac disease	1	7	Migraine	–	4
Congenital cytomegalovirus			Mitochondrial disease	–	1
Congenital heart disease	–	1	Oropharyngeal dysphagia	–	1
Congenital malformations	1	1	Osteoporosis	–	2
Congenital neutropenia	–	1	Physical disability	–	1
Connective tissue disorder	–	1	Polycystic ovary syndrome	–	3
Cystic fibrosis	5	7	POTS	4	6
Di George syndrome	–	1	Psoriasis	–	1
Down syndrome	–	1	Renal failure	–	1
Duchenne muscular dystrophy	–	1	Rhombencephalosynapsis	–	1
Ehlers–Danlos syndrome	6	12	Scoliosis	1	2
Eosinophilic oesophagitis	1	4	Sjogren's disease	1	2
Epilepsy	4	2	Spinal cord injury	–	1
Epileptic encephalopathy	1	–	Spinal muscular atrophy	1	–
Factor V Leiden	–	1	Syringomyelia	1	–
Fanconi's syndrome	–	1	Tethered spinal cord syndrome	1	–
Fatty liver disease	–	1	Thyroid disorder	–	2
Foetal alcohol spectrum disorder	–	1	Triple A syndrome	1	1
Fibromyalgia, complex and chronic pain	7	14	VACTERL association	–	1
Functional gut disorder	–	1	Vision loss	–	1
Functional neurological disorder	–	1			

Abbreviations: ARFID, avoidant restrictive food intake disorder; POTS, postural orthostatic tachycardia syndrome; S1, stage 1; S2, stage 2.

### 3.1 Stages 1 and 2

In stage 1, 456 questions were submitted, which were coded and collapsed into 40 themes. A list of the collapsed themes from stage 1 is shown in Table 4; the 20 highest‐ranked themes following stage 2 are marked with an asterisk. Following discussion at the workshop, several of these themes were collapsed further into 12 themes, and based on participant feedback, an additional theme was added for a total of 13 themes. The additional theme was ‘How can we promote and respect young people's agency and self‐advocacy?’ At the workshop, participants discussed at length the language used to capture their experience appropriately and respectfully.

**Table 4:** Collapsed themes from stage 1 that were presented for ranking in stage 2 (not listed in order of priority).

No.	Theme
A*	How can young people with chronic conditions and disabilities access treatment and support quickly and easily? E.g., Reducing waiting times, choosing from a range of options and specialists and having access to services before diagnosis.
B	How can we support young people to communicate about their chronic conditions and disabilities? E.g., Communicating in relationships, when and how to disclose diagnoses and managing online communication.
C	How can we support the identity development of young people with chronic conditions and disabilities? E.g., Increasing feelings of acceptance amongst young people and their families, acknowledging young people's feelings of being different from their peers.
D*	How can we increase access to research‐informed evidence and clinical trials for young people with chronic conditions and disabilities and their families? E.g., Techniques to help health practitioners communicate health information, tailoring information to the young person and family and clinical trial support tools.
E*	How can we support the transition of young people with chronic conditions and disabilities from paediatric to adult care? E.g., Knowing which age is best for transition, providing information to prepare young people and their families, providing mental health support and making adult hospitals less scary.
F	How can we help parents reduce career disturbance when they have a child with chronic conditions and disabilities? E.g., Due to work missed to attend medical appointments, the need to work part‐time or loss of income.
G*	How can the social exclusion of young people with chronic conditions and disabilities be minimised? E.g., Education to reduce stigma, programmes to reduce bullying.
H*	How can we help young people with chronic conditions and disabilities plan for the future? E.g., Supporting the transition to adulthood.
I*	How can young people manage the uncertainty surrounding their chronic conditions and disabilities? E.g., Sustaining hope when the future is unpredictable, managing fluctuating needs and responding to worsening or possible complications of their chronic conditions and disabilities.
J*	How can we minimise the impact of chronic conditions and disability on young people's work and study? E.g., Career options and workplace support.
K*	How can we support young people to manage their chronic conditions and disabilities at school and university? E.g., Education and training for school staff, increasing flexibility and providing support for catchup.
L	How can we reduce the impact of a young person's chronic conditions and disabilities on other children in the family? E.g., the Impact on family dynamics and functioning, the impact on relationships between parents and the balancing of time and resources between children.
M*	How can we raise awareness and community acceptance of chronic conditions and disabilities? E.g., Educating the public, teachers, family members, professionals and peers; including education about chronic conditions and disabilities in schools; identifying the best language to use in education.
N	How can families and young people find chronic conditions and disabilities advocacy opportunities and organisations to work with?
O*	How can we improve the effectiveness of treatments for chronic conditions and disabilities? E.g., Developing our understanding of best management and treatment, working to find a cure and focusing on promoting recovery.
P*	How can coordination be improved across all disciplines involved in young people's care? E.g., Coordinating advice given, sharing patient information, use of electronic records.
Q*	How can we help professionals communicate with young people and their families? E.g., Showing empathy, respecting the expertise of young people and their families, taking a nonblaming perspective and maintaining professional boundaries.
R	How can we improve sleep outcomes for young people with chronic conditions and disabilities and their families? E.g., Managing the impact of chronic conditions and disabilities on sleep and supporting carers to get enough sleep.
S*	How can we improve the process of diagnosing chronic conditions and disabilities? E.g., Making diagnosis quicker and easier, supporting young people's adjustment to diagnosis and addressing possible trauma surrounding diagnosis.
T	What causes chronic conditions and disabilities?
U*	How can we ensure treatment is appropriate for young people with chronic conditions and disabilities? E.g., Teen‐specific services, filling the gap in treatment and support for young people aged 16‐25 years.
V	How can we support and promote young people's participation in activities with their families and peers? E.g., Supporting young people and families to travel.
W	How can we empower young people with chronic conditions and disabilities to coordinate their own care? E.g., Increasing health literacy, understanding who to go to and what to do, increasing young people's capacity and confidence to manage their chronic condition and disabilities and reducing information overload.
X	How can we keep young people with chronic conditions and disabilities out of hospital and emergency departments and better understand the impact of these experiences on children and young people?
Y*	How can we ensure families are capable of supporting their children with chronic conditions and disabilities? E.g., Increasing access to medical supplies, making information more accessible and providing support services to families.
Z*	How can we support the health and well‐being of parents and families of young people with chronic conditions and disabilities? E.g., Addressing burnout, exhaustion, and physical and mental illness; provision of peer support groups facilitated by health professionals.
AA*	What are the financial support needs of young people with chronic conditions and disabilities? E.g., To address the cost of care and treatment and loss of income.
AB	How can we promote equity in diagnosis, treatment and outcomes? E.g., Understanding how socioeconomic and cultural issues impact care.
AC	How do we optimise follow‐up care and communication? E.g., Support between appointments.
AD	How can we improve the management of side effects of chronic conditions and disabilities and their treatments?
AE	How should treatment guidelines be updated and how can we ensure that health professionals act in accordance with these?
AF	How can health professionals help families follow treatment recommendations?
AG	What are the best ways to support young people who are unable to self‐manage their chronic conditions and disabilities? E.g., Residential care and home visits.
AH*	Which challenges associated with chronic conditions and disabilities impact mental health in children and young people? E.g., Coping with symptoms, presence of multiple conditions, being misunderstood, poor school performance, balancing physical and mental health needs and restrictions impacting confidence.
AI	How can we reduce the impact of challenges on the mental health of young people with chronic conditions and disabilities? E.g., Providing skills to promote better mental health.
AJ	How can we build on the strengths of young people with chronic conditions and disabilities to promote well‐being?
AK*	How can we make treatment and support more affordable? E.g., Ensuring all chronic conditions and disabilities qualify for NDIS, flexible in‐home support, a combination of professional and peer support.
AL*	How can the social isolation of young people with chronic conditions and disabilities be minimised? E.g., Peer and social support, helping friends be more inclusive, supporting healthy relationships and online communication when housebound.
AM	How can research in the area of chronic conditions and disabilities be improved? E.g., Increasing research funding, making research easier to do.
AN	How do chronic conditions and disabilities impact functioning and what support is needed? E.g., Impact on child development and cognitive function.

*Note*: The 20 highest‐ranked themes following stage 2 are marked with an asterisk.

### 3.2 Consensus workshop

The top 10 priorities following the workshop are shown in Table 5. The top‐ranked priority was *raising awareness, increasing community inclusion and reducing exclusion and isolation of young people with CCD in all aspects of life*. This priority is a composite of three themes ranked in the Top 20 themes from stage 2 of our project; conversations with the participants at our consensus workshop revealed that participants felt that the components mutually supported each other and needed to work together to achieve meaningful change. *Accessing appropriate treatment and support quickly and easily* was the second‐ranked priority, with *improving the diagnostic process* ranked third. Individual questions and reflections on these priorities revealed many barriers to access, including lack of availability of specialist services, limited understanding of specific conditions within the available services, long wait times for specialist appointments and being disbelieved or dismissed about symptoms. Further, participants discussed the difficulties in obtaining a diagnosis and the conundrum that they faced when access to services or financial support was contingent on receiving a diagnosis.

**Table 5:** Top 10 priorities for child and adolescent chronic conditions and disabilities research.

1	How can we raise awareness, increase community inclusion and reduce the exclusion and isolation of young people with chronic conditions and disabilities in all aspects of life?
2	How can young people with chronic conditions and disabilities access appropriate treatment and support quickly and easily?
3	How can we improve the process of diagnosing chronic conditions and disabilities?
4	How can we support young people living with chronic conditions and/or disabilities during school, work and study?
5	How can we improve the effectiveness of treatments and support for chronic conditions and disabilities?
6	Which challenges of chronic conditions and disabilities impact mental health in young people?
7	How can we make treatment more affordable and address the financial support needs of young people with chronic conditions and disabilities?
8	How can we promote and respect young people's agency and self‐advocacy?
9	How can we support the health and well‐being of parents, carers and families of young people living with chronic conditions and disabilities?
10	How can coordination and communication with young people and their families be improved across and between all disciplines and community services?

In describing access to support and services, young people with CCD and their parents and caregivers frequently conveyed high levels of distress, isolation and overwhelm. One parent stated: ‘Where do you go if your ONE specialist is on holidays and the GPs have no idea how to help your child? When the GPs say go to hospital then you go there, and they say wait for the specialist to return from holidays in three weeks. When your kid can't breathe or move. Or when your kid is vomiting every day for 11 months and no one has a clue what to do. Where do we go when this happens?’ (coded as ‘How can young people with chronic conditions and disabilities access appropriate treatment quickly and easily?’).

Barriers to accessing appropriate diagnosis, treatment and support were frequently described by participants and included limited awareness about specific chronic conditions and experiences of being disbelieved or disrespected by professionals. For example: ‘My daughter wants more doctors to know about her condition, to admit when they don't know about her condition, but to learn or seek out help from other medical professionals who know more. When did it become the norm for doctors to have to know everything? No one can know everything about every condition. Either listen to my daughter who has lived with it or liaise with other medical professionals who have more experience in that area’. This was coded as ‘How can we help professionals communicate with young people and their families?’ However, it also overlaps with priorities regarding *raising awareness* and *supporting self‐advocacy* and has clear implications for accessing diagnosis, treatment and support.

Priorities 4–10 encompassed appropriate support for school, work and study (priority 4); improving treatment effectiveness (priority 5); understanding mental health impacts (priority 6); improving treatment affordability and financial support (priority 7); promoting agency and self‐advocacy (priority 8); supporting the well‐being of parents, carers and families (priority 9) and improving care coordination and communication (priority 10). Additional priorities on the list that fell outside of the top 10 were (11) *How can we help young people with CCD manage uncertainty and plan for the future?;* (12) *How can we support the transition of young people with CCD from paediatric to adult care?* and (13) *How can we increase access to research‐informed evidence and clinical trials for young people with CCD and their families?*

### 3.3 Additional themes

In addition to the priorities that emerged from the workshop, an additional theme emerged through the survey responses and workshop discussions. Respondents highlighted the different types of burdens experienced by young people with invisible disabilities and the burden of ‘proof’ that young people face when requesting accommodation or support. For example, one young person wrote: ‘Teaching staff are more understanding of disease they can see (i.e., they will be fine with someone with a broken leg having a swivel chair, but they get upset when I have one because they can't see my pain). The evidence these institutions (secondary and tertiary) require to prove there is something wrong with you and that you need help is burdensome and expensive and detracts from the time my medical team could spend on helping me get better because they are forever filling out forms the university needs so that I can get the right chair, or be allowed to take medicine in lectures’. This was coded as ‘How can we support young people to manage their chronic conditions and disabilities at school and university?’ However, this theme does not adequately reflect the participant's emphasis on the different experiences of those with visible and invisible disabilities, nor the need to avoid placing a further burden on young people and their treating professionals to secure appropriate support.

### 3.4 Workshop feedback

In reflecting on the workshop, participants reported feeling satisfied with the outcomes and enjoying the process. One professional said ‘I liked the engagement with all participants in the group. Having participants with lived experience was crucial also’, while a young person stated, ‘I think it was enlightening to hear from all different perspectives ‐ I found listening to their experiences and opinions was something I would not be able to experience in my normal life’. Some participants described having a change in perspective from listening to the stories of others (e.g., one young person described placing higher importance on the priority of family well‐being after hearing a parent/caregiver describe their experience). Participants at the workshop expressed satisfaction that the final list adequately captured their priorities. However, they also acknowledged that these priorities may not reflect the priorities of certain groups, including Aboriginal and culturally and linguistically diverse families, children and young people in out‐of‐home care and children and young people who have Intellectual Disability, are neurodivergent and those who have ‘full care needs’. Participants acknowledged that while all priorities were relevant to people living with CCD, the order of importance may vary depending on one's age, cultural background or the type of CCD experienced.

To improve the workshop, participants recommended allowing more time and suggested that the workshop be conducted across two separate sessions, given that the target group is prone to fatigue and other symptoms that may limit their participation. It was suggested that the use of polling software would enhance the online workshop experience, particularly for those who struggled to keep up with the pace of conversation and multiple participants speaking at the same time. Further, participants highlighted the need to make use of the chat function for those who had low bandwidth internet access and to provide young people with vouchers to purchase internet data access ahead of time.

When asked for their thoughts about how the number 1 priority should be addressed, several participants emphasised the need to change the narrative of CCD being seen as a personal failure. One young person stated ‘We need to adopt and promote the social model of disability, where our conditions are not viewed as personal failings, but a failure of society to accommodate for everyone…… If we can be promoted as being as strong and valued as others, then we will become more accepted’. Additionally, several participants highlighted the importance of paying attention to the language used to describe CCD: ‘I believe using correct terms for chronic conditions and/or disabilities is key to greater understanding which in turn leads to reduced exclusion’. Young people highlighted the need to acknowledge that systematic ableism—that is, discrimination and stigma against people with CCD often goes unnoticed: ‘even when you have lived experience, sometimes you don't even question it because it's so entrenched’.

Participants described the need to change representations of CCD in the media, by reducing the use of ‘exaggerated’ representations, increasing the visibility of young people and professionals with lived experience and ‘making the invisible, visible’. Others highlighted the need for awareness‐raising through ‘funding to produce appealing information, materials and campaigns to educate and raise the profile’ and ‘education on the impact of chronic illness outside of the illness itself. It is much broader and investing in impactful strategies will improve outcomes in the longer term’. Several participants described a need to increase empathy towards those with chronic conditions, highlighting that public dialogue about COVID‐19 ‘only’ leading to mortality in people with ‘pre‐existing conditions’ had made them feel dispensable and devalued. Participants also highlighted the need for government recognition and support, particularly considering the disproportionate impact of COVID‐19 on young people with CCD. Additionally, they called for a reduction in ‘bureaucratic silos’ and more collaboration across sectors.

Young people and parents described the need for basic accessibility at community events and places ‘so that it isn't a big deal for people with disabilities/chronic conditions to be there’, as well as sports and recreational activities specifically for people with CCD. Several participants recommended having a liaison person who can advocate on behalf of the young person in school, work and health environments. Additionally, participants recommended that workplaces need to become more inclusive, by adapting current policies and making roles available specifically for those with CCD. One participant highlighted that diversity and inclusion initiatives often fall short of including those with CCD: ‘Many of these institutions promote diversity but do not live up to it, so it feels surface level. Especially if they are trying to address disadvantaged groups but then cherry‐pick a few characteristics (which are all important….but chronic conditions and disability seems to be the one they avoid the most). I know in terms of medicine/doctors, the best ones out there would understand your experiences firsthand in some way ‐ it would be very refreshing to see a GP or specialist who actually has lived experience!’

## 4 DISCUSSION

The aim of this study was to prioritise research questions for childhood CCD research by integrating perspectives of children and young people with CCD, their parents/caregivers and professionals working in the field. Priority‐setting processes with specific CCD have often reflected a biomedical or clinically focused approach to CCD.^29^ While these perspectives were also reflected in the current process—in priorities such as *improving diagnosis*, *increasing treatment access* and *improving effective treatments*, findings from the current project also align with a social model in which attention is paid to the ways in which impairments are a product of the environment rather than of the individual. The top 10 priorities provide guidance on key focus areas to guide research, translation and advocacy approaches. The complexities of the stories behind the priorities highlight the need to work alongside young people and other stakeholders with lived experience to design research and derive solutions to address these questions. The findings also highlight the need to consider the different experiences of those with visible and invisible disabilities and the need to avoid placing a further burden on young people and their families in addressing the priorities that have emerged. Reflections on the workshop process also provide valuable guidance for how online priority‐setting processes can be improved for young people living with CCD.

### 4.1 Awareness and inclusion

Prior work with young people living with CCD has highlighted the role of stigma, prejudice and ableist expectations in experiences of disability, inclusion and exclusion.^31^, ^32^ Moreover, prior research has found that in some cases, children (including those with disability) actively participate in ableist discourses,^33^ including comparing the severity of impairment across different types of illness and disability.^33^, ^34^ Our participants described ways in which exclusion and isolation can happen across all settings that young people and their families interface with, including healthcare, school, education, social relationships and employment. While awareness‐raising programmes such as school‐based interventions have demonstrated improved knowledge and acceptance of disabilities, these interventions are often unable to address the broader social environment that perpetuates the exclusion of people living with CCD.^35^ There is a need to better understand how structural and individual factors within these systems contribute to exclusion and how these can be changed to create more inclusive environments. Additionally, there is a need to better understand how ableist attitudes might become internalised amongst young people living with disability, thereby contributing to mental health problems and potentially undermining participation across different life domains.^36^

### 4.2 Improving the accessibility, efficiency and effectiveness of diagnosis, treatment and support

Our second‐ and third‐ranked priorities are related to improving efficiency and reducing the burden of diagnostic and treatment processes. Prior work has demonstrated that people with chronic illnesses frequently report negative healthcare experiences, including being dismissed.^37^ These experiences each contribute to treatment burden, described as ‘the “work” of living with a chronic condition’,^38^ which has been linked to poor health and employment outcomes. It is noteworthy that, young people, particularly those with multiple chronic conditions, are at heightened risk for experiencing treatment burden.^36^ These findings emphasise the need for capacity‐building with healthcare professionals, as well as *supporting agency and self‐advocacy in young people*, which was our sixth priority. Building health professional capacity and supporting agency in people with CCD and their families should be supported by a system‐level change to improve patient experience within the hospital system and integrate with primary care. This also aligns with the fifth‐ranked priority of improving treatment and support. While measures of ‘treatment effectiveness’ may vary across different CCDs, this priority also highlights the need for a better understanding of which patient‐reported outcomes are considered most meaningful for young people with CDD, particularly from a transdiagnostic perspective.

### 4.3 School and employment

Our findings reveal a need for more research and policy and practice change focused on ensuring that young people with CCD can participate equitably in school and employment settings. Participants reported myriad adverse experiences in school and work settings, including (a) education staff and employer's lack of awareness about the impact of CCD; (b) exclusion of people with CCD from certain occupational settings; (c) difficulty accessing required accommodations to participate equitably in school and work; (d) impact of symptoms or treatment side‐effects on cognitive capacity; (e) disruption to school or employment due to hospitalisation and (f) bullying and difficulties forming or maintaining social networks at school. Research from outside of Australia has demonstrated effective strategies for schools to better engage children and young people with chronic conditions, such as the use of hybrid‐virtual classrooms, where young people with chronic conditions can participate in class activities in real‐time via online conferences.^39^ Employing such practices within Australian schools may simultaneously reduce the education disruption experienced by children and young people with chronic conditions while facilitating their ongoing connections to peers.^40^ Accordingly, research partnerships with Australian schools trialling hybrid‐virtual classrooms and accompanying strategies are necessary to justify policy change and broader implementation of engagement strategies.

### 4.4 Understanding mental health outcomes

When compared to their peers, young people living with chronic conditions are disproportionately impacted by mental health disorders such as depression and anxiety.^41^ Participants in the current study called for a greater understanding of how CCD impacts mental health. While mental health interventions for people with CCD seem to have demonstrated some positive impact, findings have ultimately been conflicting.^42^ Further, there is growing evidence to suggest that the documented mental health discrepancy is not inherently due to the presence of chronic conditions or disability but is in fact a product of the social context.^43^ For example, Emerson et al.^44^ demonstrated that in a nationally representative sample of Australians aged 15–29 years, the link between disability and subjective well‐being was almost entirely accounted for by the experience of adversity and relative lack of access to social and economic resources. These findings further indicate the need for future research to consider the interaction between individual and social characteristics of CCD when exploring mental health impacts.

### 4.5 Strengths and limitations

Promoting children's voices, agency and competency and protecting their rights are critical in childhood CCD research.^45^ The current study had both strengths and weaknesses in this regard. The strengths of the research include the involvement of children, young people and family members with lived experience of a broad range of CCD. This brings a diversity of perspectives to the process and helped us to identify priorities that are likely relevant for many children, young people and family members. Relative to prior CCD priority‐setting work, we also included a large proportion of young people and parents/caregivers in our sample. This helps to increase confidence that the priorities identified are reflective of those with lived experience. Confirmation of these priorities with an independent sample would help to determine their generalisability.

We also acknowledge that there are many young people who would not have been able to participate in the research activities outlined in this study without additional support. Because we used a convenience sample, we are unable to determine how representative our sample is, or which young people were excluded from the process. Further, intersectionality was not measured in our study and young people with intersectional identities likely face additional challenges and may have different priorities. For example, prior work emphasises the need for specific consideration to be given to promoting access for Aboriginal and Torres Strait Islander families, who frequently experience intersectional disadvantage.^46^ Based on recommendations from our Advisory Groups, future research should focus specifically on the priorities of Aboriginal and Torres Strait Islander children and young people with chronic conditions to ensure that their perspectives are appropriately represented. We did not seek to achieve this in the current study, based on advice from our Aboriginal Research and Development Unit that such engagement could not be appropriately achieved within our budget and timeframe.

## 5 CONCLUSIONS

The current priorities, derived from a robust sample of children and young people with lived experience, their parents and caregivers and the professionals who work with them, highlight the interplay between social and individual facets of the CCD experience. They demonstrate the need to consider multiple aspects of CCD impact when designing research that is meaningful to those with CCD and their support networks. Future work should focus on the involvement of those with lived experience in designing research to address these priorities, as well as confirmation of these priorities with independent samples, including specific subgroups.

## AUTHOR CONTRIBUTIONS

Amy Finlay‐Jones conceived the study with the support of Belinda Frank and Anne McKenzie and secured funding with Julie Dart, Elizabeth Davis, Anne McKenzie and Keely Bebbington. Amy Finlay‐Jones, Karina Prentice, Rebecca Sampson, Jacinta Freeman, Amber Bates, Belinda Frank, Keely Bebbington and Anne McKenzie assisted with data collection and Amy Finlay‐Jones, Karina Prentice and Rebecca Sampson assisted with data analysis. All authors assisted with data interpretation. Amy Finlay‐Jones, Asha Parkinson, Karina Prentice and Jayden Lucas drafted the manuscript. All authors reviewed the manuscript.

## CONFLICT OF INTEREST STATEMENT

The authors declare no conflict of interest.

## ETHICS STATEMENT

Ethics approval was provided by the Government of Western Australia Child and Adolescent Health Service Human Research Ethics Committee (RGS000000003842). All participants provided informed consent to participate in each stage of the project. All participants provided informed consent for deidentified data to be published.

## Data Availability

Data are not publicly available as ethics approval was not granted for data sharing.
